# Palliative Care Rounds: A Thanksgiving Break to Discuss Rib Fractures

**DOI:** 10.7759/cureus.52396

**Published:** 2024-01-16

**Authors:** Richard V Guthrie, Sharene Best, Ryan Baldeo, Jamie Ellis-Wittenhagen, Tyler Murphy

**Affiliations:** 1 Division of Palliative Medicine, Department of Medicine, Mayo Clinic Arizona, Phoenix, USA; 2 Division of Hematology/Oncology, Department of Medicine, Mayo Clinic Arizona, Phoenix, USA

**Keywords:** patient history, thorough physical examination, opioids analgesia, integration of palliative care service, patient-doctor communication, multi-modality pain management, pathological rib fracture

## Abstract

Rib fractures are common injuries, especially in the frail and elderly. They can happen in isolation or may be associated with significant concomitant morbidity, including but not limited to pain, pneumonia, or pneumothorax. In the palliative care population, rib fractures can be overlooked or attributed to other entities, which may lead to inappropriate treatment. The commonly accepted standards of care for the treatment of rib fractures are centered around early and adequate pain control, and stabilization of other complications. Accurate diagnosis and management demand a thorough history and physical examination, effective communication, and intentional clinical consideration of all differential diagnoses.

## Introduction

Rib fractures are common injuries. It has been reported that over 350,000 adults in the United States were diagnosed with rib fractures in 2017 [1]. These injuries are most often caused by blunt trauma to the chest, but can also result from severe coughing, athletic movements, or occult ways. They may happen in isolation, or be associated with significant morbidity, including pain, pneumothorax, pulmonary laceration, or pneumonia. Risk factors include osteoporosis, sports participation, and malignant lesions in a rib [2]. Morbidity increases with increasing age and number of fractures [3].

In the palliative care population, rib fractures can be easily overlooked by focusing on other etiologies that might involve pain, including infection, tumor discomfort, pleural effusions, or other complications of cancer and cancer treatment. Missed or delayed diagnosis can result in a misguided treatment plan for the actual pain etiology.

## Case presentation

The patient was a 93-year-old female with a history of fallopian tube and breast cancers more than five years prior, both definitively treated without recurrence. More recent oncologic history included diagnosis of left lung adenocarcinoma, within six months prior to presentation. The patient elected to receive only radiation therapy to the primary lesion, forgoing systemic treatment. In total, she received four fractions to the left lower lung lobe; the last treatment was four months prior to presentation.

The patient was admitted to the hospital with left-sided chest and flank pain for one day. The patient initially reported that the pain started somewhat insidiously and was noticed upon awakening one morning. It was characterized as sharp, with radiation toward the midline at the level of the mid-thoracic spine. Pain severity was 5/10 while sitting up, and 10/10 when lying down flat on her back. Acetaminophen 500 mg was ineffective, and oxycodone 5 mg that she had at home, left over from a previous outpatient procedure, also did not help.

On exam, the primary team described an elderly patient in mild distress, clutching her left side, and occasionally shouting out in pain with almost any movement. Vital signs were normal except for mild tachypnea at 22 breaths per minute. Physical exam was otherwise remarkable for a 4/6 systolic ejection murmur at the right upper sternal border consistent with a known history of aortic stenosis and left-sided thoracic and upper abdominal tenderness to palpation that did not cross the midline in the front or back. There was no rash.

**Figure 1:**
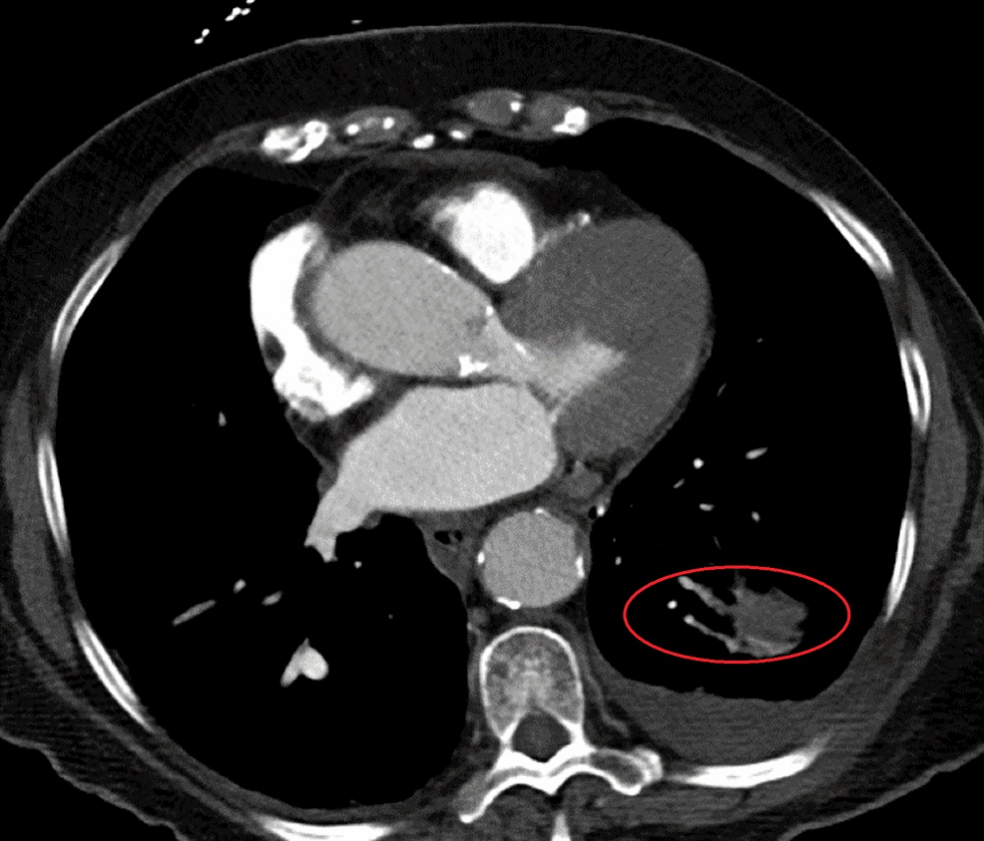
High-resolution coronal CT image demonstrating bronchial thickening and traction bronchiectasis.

Laboratory studies were within normal limits, except for findings consistent with her known chronic kidney disease stage 3. Urinalysis raised concern for UTI. A portable chest X-ray suggested atelectasis. There was no consolidation or pleural effusion seen on the imaging. Computed tomography of the chest corroborated plain film findings and noted bronchial thickening and traction bronchiectasis. Please see Figure 1 below in which these findings are demonstrated.

The patient was admitted for suspicion of UTI, shingles, and pneumonia. She was treated with broad-spectrum antibiotics to cover urinary and pulmonary pathogens, and antivirals to cover shingles. She was also started on conservative scheduled acetaminophen 500 mg every six hours, as well as oral (oxycodone 5 mg every six hours as needed) and parenteral (hydromorphone 0.5 mg every four hours as needed) opioids for breakthrough pain.

**Figure 2:**
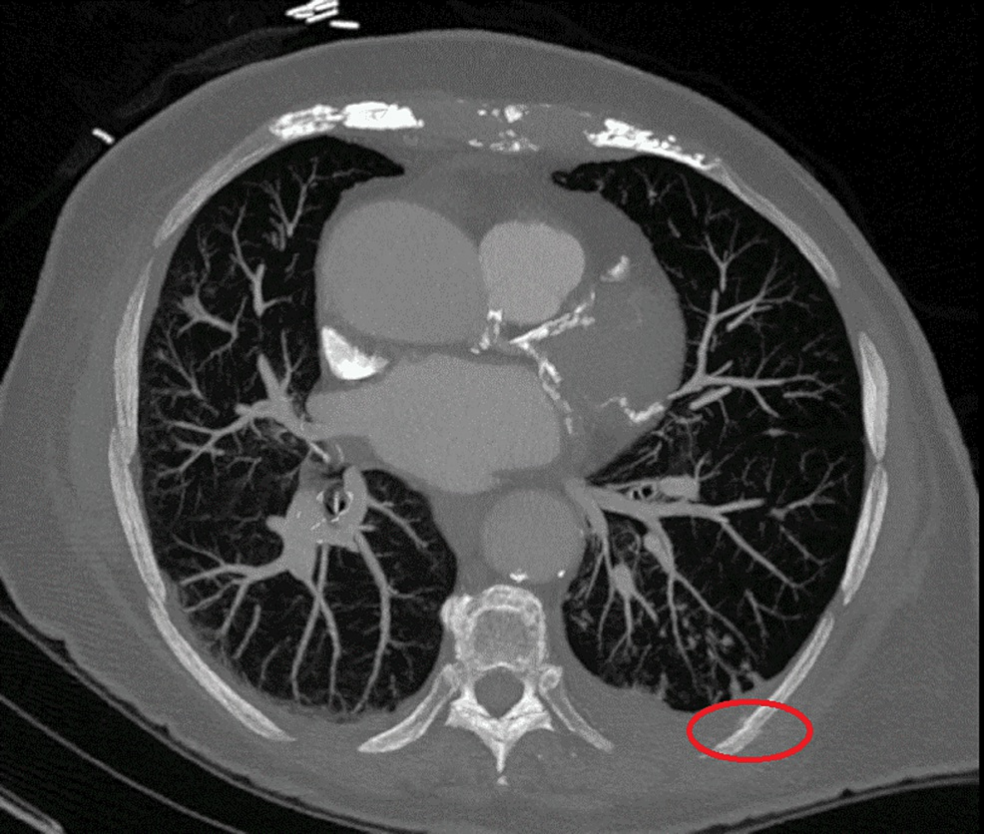
High-resolution coronal CT image demonstrating fracture at left rib 8.

**Figure 3:**
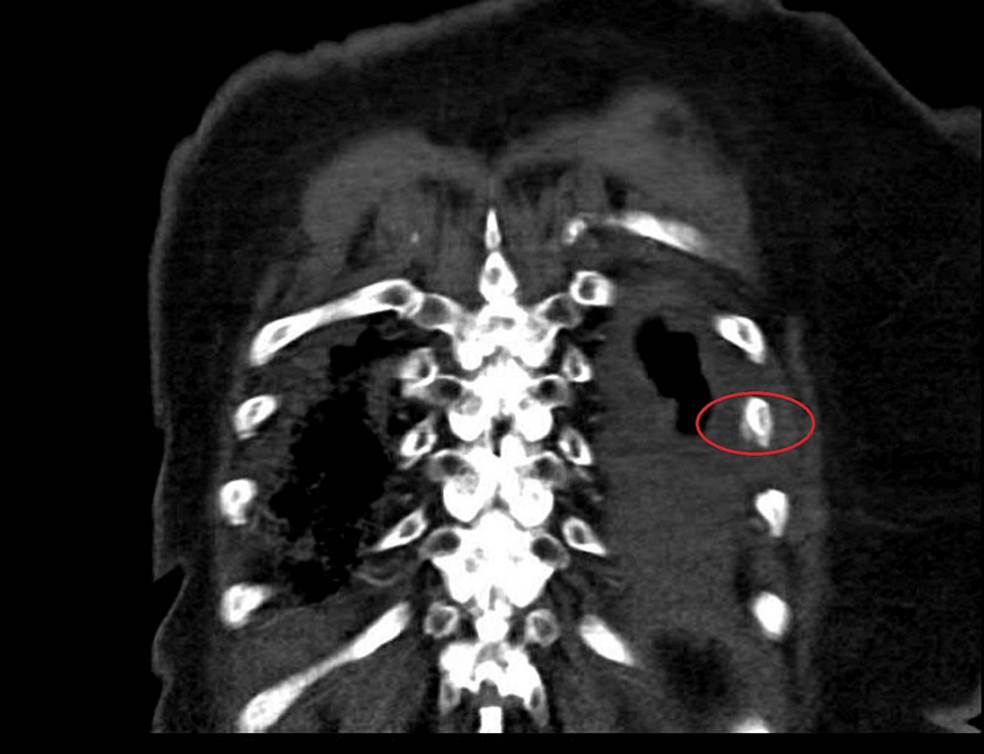
High-resolution axial CT image showing cortical disruption of left lateral rib 8.

Our palliative care team was consulted on hospital day three, which happened to be Thanksgiving. A more detailed pain history revealed that her pain actually started days prior to arrival but got significantly worse, suddenly, upon sitting up in bed the morning of the presentation. The patient felt as if “something [was] catching back there.” Upon reviewing the plain chest radiographs and CT angiogram (CTA), our team queried the possibility of left-sided rib fracture(s), which would have been consistent with her expanded history. We reviewed the images with our radiologists, who confirmed our suspected diagnosis of fractures in left ribs 8 and 9. The CTA report was amended accordingly. Please see Figure 2 (coronal image) and Figure 3 (axial image) below, in which the red ovals denote the subtle fractures we discovered. Though this may have been considered unfavorable news, our patient was relieved to know that the cause for her pain had been found. The patient “thought she was going crazy,” and assumed that she was not being taken seriously, because she was being treated for entities that she perceived to be unrelated to her chief complaint of pain. Instead, we helped her find a “reason to be thankful.”

Upon confirming this diagnosis, we altered the analgesic regimen to include optimizing scheduled acetaminophen at 1,000 mg three times daily, continued her home dose of pregabalin at 50 mg twice daily, and added a 5% lidocaine patch with the standard cadence of 12 hours on/12 hours off. We also provided recommendations for nonpharmacologic methods, including ice packs, and mindfulness exercises, including meditation and deep breathing as able in the form of incentive spirometry, which is standard of care in the management of rib fractures. Additionally, we consulted our colleagues in pain medicine who performed injections of the left subacromial bursa and subdeltoid. Though not seemingly anatomically related, these procedures did benefit the pain stemming from her rib fractures. The patient was discharged home the next day, feeling much better overall.

## Discussion

This case highlights two fundamental skills for which we all strive toward excellence in everyday practice: history and physical examination, and effective communication. By taking the time to collect a more detailed account of her pain, and revisit the imaging studies, we were able to identify the true etiology of her pain and treat it effectively with an appropriate multimodal approach that did not include opioids. Opioids are not recommended as initial management of pain related to rib fractures.

As we know, patient satisfaction is at the forefront of our healthcare environment today. Evidence consistently shows that good communication is what patients desire most [4,5]. They want teams who listen attentively, explain medical issues in simple language, include them in decision-making, and communicate effectively with other teams. They also wish for their families to feel heard and respected, and to leave encounters with clear instructions and a commitment to partnership. Our patient repeatedly expressed gratitude for the involvement of our team, especially for the extra time spent exploring her history, and the multidisciplinary collaboration our team promoted in her management.

Early and adequate pain control remains the cornerstone of rib fracture management and can mitigate further complications, including splinting and subsequent atelectasis and/or pneumonia. Treatment of choice depends on many factors, including patient experience with analgesics, clinician comfort levels, and the availability of interventional methods. Nonsteroidal anti-inflammatory drugs (NSAIDs) are a common first-line treatment, unless contraindicated, as in our patient with renal impairment. The next line of treatment can include acetaminophen, lidocaine or other transdermal patches, and heat and/or ice. Early management of rib fractures should also include respiratory care. Incentive spirometry (IS) has been shown to be a valuable aspect of this regimen [1]. In a study of 50 patients with traumatic rib fractures, IS reduced pulmonary complications and improved pulmonary function test results [6].

If these treatments are found to be ineffective, opioids can be considered while carefully weighing risks and benefits. In our patient, given her other comorbidities and advanced age, avoiding opioids was preferable. Opioids in the elderly and hospitalized patients can lead to increased rates of delirium in addition to other common side effects such as constipation [7]. An accurate diagnosis initially for our patient would have likely afforded conservative, opioid-sparing management.

## Conclusions

This case exemplifies the importance of effective communication. This often includes performing a more robust history and physical examination in the interest of providing comprehensive care and facilitating a more accurate diagnosis and treatment, acknowledging the challenges presented by confounding differential diagnoses. It also validates the many benefits of striving for expertise in collaboration and coordination of care. Our suspicion for rib fractures was heightened when we spent more time with the patient exploring her pain history. By working together with our colleagues in radiology, pain medicine, and hospital internal medicine, we were able to achieve a good outcome for the patient and to nurture our collaborative relationships with those other services.
